# Losartan, an angiotensin-II type 1 receptor blocker, attenuates the liver fibrosis development of non-alcoholic steatohepatitis in the rat

**DOI:** 10.1186/1756-0500-2-70

**Published:** 2009-05-05

**Authors:** Hitoshi Yoshiji, Ryuichi Noguchi, Yasuhide Ikenaka, Tadashi Namisaki, Mitsuteru Kitade, Kosuke Kaji, Yusaku Shirai, Junichi Yoshii, Koji Yanase, Masaharu Yamazaki, Tatsuhiro Tsujimoto, Hideto Kawaratani, Takemi Akahane, Yosuke Aihara, Hiroshi Fukui

**Affiliations:** 1 Third Department of Internal Medicine, Nara Medical University, Shijo-cho 840, Kashihara, Nara 634-8522, Japan

## Abstract

**Background:**

Apart from simple steatosis, the non-alcoholic steatohepatitis (NASH) can progress into liver fibrosis and cirrhosis. To date, however, no widely accepted therapeutic modalities have been established against NASH in the clinical practice. To find out promising new therapeutic agents, it is important to employ an appropriate experimental model of NASH, such as association with insulin resistance.

**Findings:**

In the current study, we found that losartan, a clinically used angiotensin-II type 1 receptor blocker, significantly attenuated a choline-deficient L-amino acid-defined (CDAA) diet-induced steatohepatitis in obese diabetic- and insulin resistance-associated Otsuka Long-Evans Tokushima Fatty (OLETF) rats. The transforming growth factor-beta, a well-known major fibrogenic cytokine, was also suppressed in a similar magnitude to that of the fibrosis area. Noteworthy was the finding that these inhibitory effects were achieved even at a clinically comparable low dose.

**Conclusion:**

Since losartan is widely used without serious side effects in the clinical practice, this agent may be an effective new therapeutic strategy against NASH.

## Findings

The spectrum of non-alcoholic fatty liver diseases (NAFLD) ranges from simple steatosis to cirrhosis. Whereas simple steatosis seems to be a benign and non-progressive condition, non-alcoholic steatohepatitis (NASH) is recognized as a potentially progressive disease that may cause cirrhosis, an end-stage liver disease, and hepatocellular carcinoma (HCC) [1,2]. The patients with NAFLD frequently have many clinical complications, such as obesity, type 2 diabetes mellitus, and insulin resistance [3]. While sustained weight loss should be very effective to improve NAFLD, it is somewhat difficult for many patients to change their life style. Accordingly, efforts are currently directed worldwide at overcoming NAFLD, especially NASH. Since insulin resistance is nearly universal in the patients with NASH, and it plays a pivotal role in the pathogenesis of NASH, many studies attempted to employ insulin sensitizer as a therapeutic modality against NASH. Although pioglitazone, a selective peroxisome proliferator-activated receptor gamma agonist, has shown some beneficial effects in the patients with NASH [3], there are still several unsolved questions. Since a long-term treatment is required to maintain the therapeutic benefits, the long-term safety of these drugs in the patients with chronic liver diseases should be proven. Another member of thiazolidinedione (TZD) class; namely, triglitazone caused fulminant hepatitis in several patients. Moreover, recent studies have questioned the long-term safety of TZD, especially rosiglitazone. Furthermore, it has been reported that TZD alone without lifestyle alternation may not achieve the anticipated clinical benefit [4]. Collectively, some time may be still required until the common application of TZD, including pioglitazone, for the treatment of NASH in the clinical practice.

**Figure 1:**
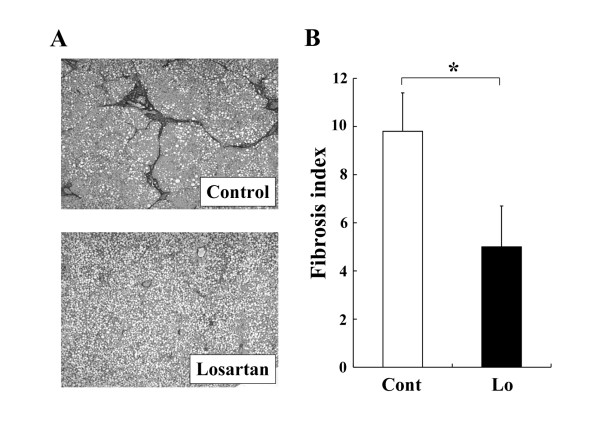
**Effect of losartan on the liver fibrosis development in the OLETF rats**. (A): Microphotographs of the liver of CDAA-treated OLETF rats. (B): The fibrosis area was evaluated by an image-analyzer. **Losartan (30 mg/kg/day) **significantly suppressed the CDAA-induced liver fibrosis development in the OLETF rats. Noteworthy was the finding that this inhibitory effect was achieved even at a clinically comparable low dose. The data represent the mean ± SD (n = 5). *: Statistically significance between the indicated group (p < 0.01).

**Figure 2:**
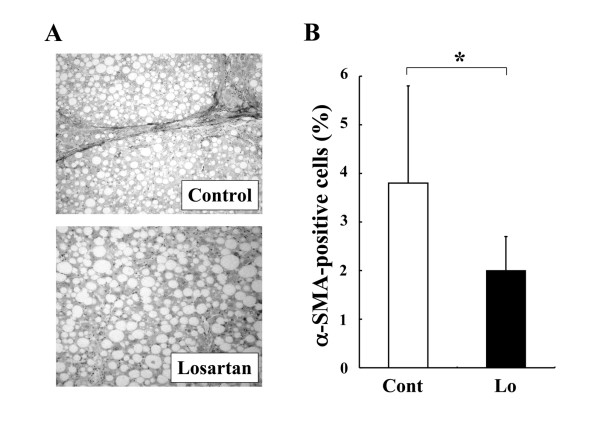
**Effect of losartan (30 mg/kg/day) on the activated hepatic stellate cells in the OLETF rats**. (A): Representative features of α-SMA-positive activated hepatic stellate cells in the liver of CDAA-treated OLETF rats. (B): Densitometric analysis of α-SMA-positive cells. The α-SMA-positive cells were significantly suppressed by treatment with losartan almost in parallel with reduction of liver fibrosis development. The data represent the mean ± SD (n = 5). *: Statistically significance between the indicated group (p < 0.01).

The renin-angiotensin system (RAS) reportedly plays an important role in insulin resistance, and suppression of angiotensin-II (AT-II) ameliorates insulin resistance [5]. We and other group have shown that suppression of AT-II by the clinically used angiotensin-converting enzyme inhibitor (ACE-I) and AT-II type 1 receptor blocker (ARB) significantly attenuated the liver fibrosis development along with inhibition of the activated hepatic stellate cells (HSC) [6]. A choline-deficient, amino acid-defined (CDAA) diet induces histological changes similar to those of the human NASH. It has been reported that ACE-I and ARB markedly attenuated the CDAA-induced liver fibrosis development along with suppression of the activated HSC [7,8]. However, a downside of the CDAA model is that it does not exhibit several common features of NASH, such as insulin resistance and diabetes mellitus. To examine the precise pharmacological action of any drug, it is important to examine its therapeutic effect under the condition of insulin resistance. In the current study, we found that losartan, an ARB, significantly suppressed the CDAA-induced liver fibrosis development in the Otsuka Long-Evans Tokushima fatty (OLETF) rats, which commonly have obesity, diabetes mellitus, and insulin resistance (Fig. 1). The total experimental period was 12 weeks. The rats received losartan daily in the drinking water (30 mg/kg/day) for 8 weeks from week 4. The concentration of losartan in the drinking water was adjusted according to the water intake to maintain a constant daily dose of the drug. This dose was almost comparable to that used in the clinical practice as described previously [9]. Losartan treatment did not cause alterantion of the serum ALT level, indicating that the losartan did not cause hepatotoxicity, and the inhibitory effect of losartan was not a secondary response to a cytoprotection effect of this agent against CDAA-induced liver injury. Neither another several serum markers such as total cholesterol and total bilirubin were affected by losartan (Table 1). We next carried out the immunohistochemical analysis of α-SMA to examine the effect of losartan on hepatic stellate cells (HSC) activation during liver fibrosis development. The inhibitory effect of losartan on α-SMA exerted most parallel reduction (Fig. 2). The serum level of TGF-beta, that was mainly produced in the activated HSC, also suppressed by treatment of losartan at similar magnitude either (Table 1). These results suggested that the anti-fibrotic effect of losartan was achieved by suppression of HSC activation. Azan-Mallory staining was employed for determination of the liver fibrosis development, and the semi-quantitative analysis of fibrosis development and immunopositive cell area were carried out with a Fiji-BAS 2000 image analyzing system (Fuji, Tokyo, Japan) as described previously [8].

**Table 1:** Effect of Losartan on several markers of the OLETF rats

	OLETF	OLETF+Losartan
Body weight (g)	671.3 ± 35.2	662 ± 41.1

Liver weight (g)	25.8 ± 4.3	22.4 ± 4.0

Glucose (mg/dl)	271.3 ± 41.0	262.0 ± 36.8

Insulin (nM/ml)	128.0 ± 10.4	117.2 ± 9.6

ALT (IU/l/dl)	102.4 ± 17.8	96.7 ± 16.6

Total bilirubin (mg/dl)	0.11 ± 0.07	0.14 ± 0.08

Total cholesterol (nmol/l)	1.04 ± 0.12	0.93 ± 0.11

TGF-β (ng/mg liver)	56.6 ± 14.3	34.3 ± 10.1*

Results are expressed as means ± SD

*: Statistically significant diferences as compared with OLETF-control group

Noteworthy was the finding that this inhibitory effect was achieved even at a clinically comparable low dose. Since losartan is widely used in the clinical practice without serious side effects, this agent may be an alternative therapeutic agent against NASH. Actually, a pilot study has shown that ARB may exert beneficial effects in the patients with NASH [10]. A large-scale prospective randomized clinical trial is required in the future.

## Competing interests

The authors declare that they have no competing interests.

## Authors' contributions

HY conceived of the study, carried out the main body of the project and prepared the manuscript. RN, YI, TN, MK, KK, YS, JY, KY, MY, TT, HK, TK, YA, and HF participated the most part of the studies such as animal handling and sample analysis. All authors read and approved the final manuscript.
